# Low-dose electron microscopy imaging for beam-sensitive metal–organic frameworks

**DOI:** 10.1107/S1600576724007192

**Published:** 2024-09-05

**Authors:** Yuhang Liang, Yi Zhou

**Affiliations:** a School of Physical Science and Technology and Shanghai Key Laboratory of High-Resolution Electron Microscopy ShanghaiTech University Shanghai201210 People’s Republic of China; Uppsala University, Sweden; The European Extreme Light Infrastucture, Czechia

**Keywords:** transmission electron microscopy, low-dose imaging, metal–organic frameworks, beam sensitive materials

## Abstract

Metal–organic frameworks (MOFs) have garnered significant attention in recent years owing to their exceptional properties. Understanding the intricate relationship between the structure of a material and its properties is crucial for guiding the synthesis and application of these materials. (Scanning) Transmission electron microscopy (S)TEM imaging stands out as a powerful tool for structural characterization at the nanoscale, capable of detailing both periodic and aperiodic local structures. However, the high electron-beam sensitivity of MOFs presents substantial challenges in their structural characterization using (S)TEM. This paper summarizes the latest advancements in low-dose high-resolution (S)TEM imaging technology and its application in MOF material characterization. It covers aspects such as framework structure, defects, and surface and interface analysis, along with the distribution of guest molecules within MOFs. This review also discusses emerging technologies like electron ptychography and outlines several prospective research directions in this field.

## Abstract 2

This topical review discusses the recent progress in low-dose high-resolution imaging of metal–organic framework materials and gives a brief outlook on possible future directions.

## 1. Introduction

Metal–organic frameworks (MOFs), as a representative type of porous material, are composed of inorganic metal nodes/clusters and organic ligands (Furukawa *et al.*, 2013 ▸). Their distinctive framework structures provide a large specific surface area and high porosity, making them very promising for applications in catalysis (Zhao *et al.*, 2022 ▸), gas/water adsorption and storage (Åhlén *et al.*, 2023 ▸; Song *et al.*, 2023 ▸), chemical sensing (Wu *et al.*, 2020 ▸), and energy storage and conversion (Liu *et al.*, 2024 ▸). MOFs exhibit highly complex structures. This complexity arises from the multitude of ways in which metal nodes and organic linkers can be arranged in three-dimensional space. The intricate connectivity leads to a myriad of structural possibilities, which makes the precise determination of their crystalline structure especially challenging. Additionally, synthesizing large single crystals of MOFs poses significant problems, particularly when designing new structures with complex ligands, which often renders them unsuitable for single-crystal X-ray diffraction measurements. For microcrystalline materials, powder X-ray diffraction (PXRD) is commonly used, employing Pawley or Rietveld refinement to match with a simulated structure. However, limitations arise in PXRD due to peak overlap, which can lead to a loss of structural information. Particularly, in complex MOFs with large unit cells and unique structures, the small size of MOF nanocrystals presents further challenges to traditional structural analysis methods.

Despite the numerous difficulties, understanding the structural information is crucial for establishing the relationship between the structure and properties of MOFs. To fully exploit the potential of their diverse applications, an atomic-scale determination of their crystal structures is essential, alongside an understanding of the mechanisms underlying their structural evolution. The main factors that affect the properties of MOF materials include the surface, defects and host–guest interactions. Surfaces have an impact on surface-related properties and growth processes, and defects can be intentionally introduced under control for modification (Cliffe *et al.*, 2014 ▸) or spontaneously generated during the assembly process (Shen *et al.*, 2020 ▸), serving various applications by providing active sites for metal ions and adjusting the porosity of the MOF. As porous skeletons with high specific surface area and high porosity, MOFs offer regular and regionally large accommodations for guest species, including nanoparticles, clusters and molecules (Esken *et al.*, 2010 ▸; Li *et al.*, 2019 ▸). The controllable incorporation of guests and synergetic interactions with the host allow for diverse applications (Lu *et al.*, 2012 ▸). However, the investigation of these key microstructures with high resolution presents significant challenges.

**Figure 1:**
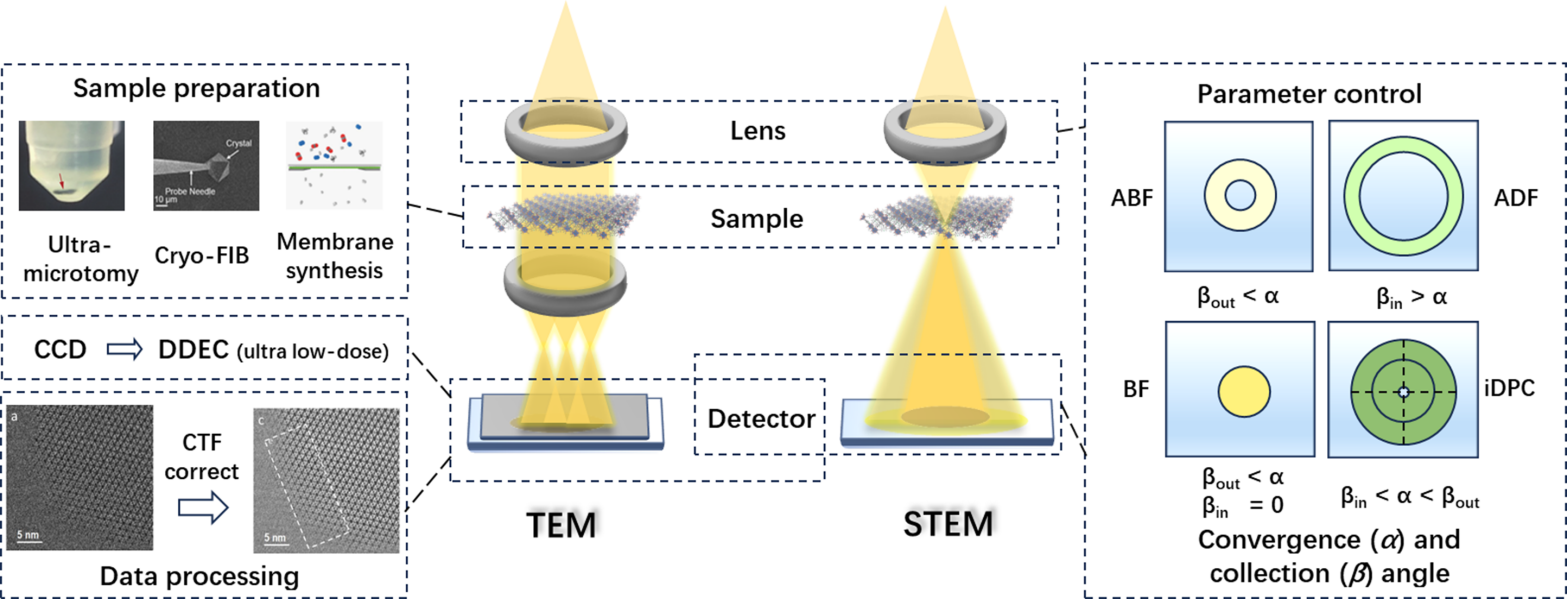
Methodology development for high-resolution imaging of MOFs in both TEM and STEM mode. Various methods for improving the image quality of MOFs are available, such as advanced sample preparation methods [reproduced from the work of Zhou *et al.* (2022 ▸) and Liu, Miao *et al.* (2023 ▸) under a Creative Commons Attribution 4.0 International License], improvement in detectors and data processing [from Zhang *et al.* (2018 ▸), reprinted with permission from AAAS], and parameter optimization.

High-resolution (scanning) transmission electron microscopy [HR-(S)TEM] imaging is extensively employed for visualizing the microstructures within MOFs, providing complementary insights beyond those obtained through diffraction methods (Xiao *et al.*, 2023 ▸). The interaction of high-energy electrons with the sample enables the direct observation of its internal structure, allowing for atomic-scale imaging of both interior and surface regions of MOFs. This encompasses the observation of both periodic and aperiodic structures, as well as local defects and porous characteristics (Zheng *et al.*, 2023 ▸). However, because of their organic component, MOFs are highly sensitive to electron beams (Wiktor *et al.*, 2017 ▸; Chen *et al.*, 2020 ▸). The traditional method to mitigate beam damage is using low voltage to reduce the bombardment of the electrons. Unfortunately, the main damage mechanism in MOFs is the radiolysis effect of the electron beam, rather than knock-on damage, and this effect is more pronounced under low voltage (Egerton, 2012 ▸, 2019 ▸; Ghosh *et al.*, 2019 ▸). Considering most of the beam damage is dose related, it is crucial to develop methods with a sufficiently low electron dose (low-dose TEM) that can capture the structure before any damage occurs (Liu *et al.*, 2020 ▸). However, by reducing the electron dose, there is a consequent deterioration in the signal-to-noise ratio of the image, leading to an inability to obtain the required structure information. While improving the sample quality through advanced preparation techniques can yield better results, this is limited to cases with a high signal-to-noise ratio. Therefore, for TEM imaging, it is optimal to acquire the image at the maximum tolerable electron dose in order to preserve structure details. To address the issue of maximum electron dose that can be tolerated by MOFs, Zhu *et al.* (2017 ▸) provided an explanation by monitoring the disappearance of high-resolution electron diffraction spots in ZIF-8 crystals under cumulative electron doses. At an electron dose rate of 1 e^−^ Å ^−2^ s^−1^, the high-resolution diffraction spot of the crystal at 1.7 Å started to vanish at a cumulative dose of 25 e^−^ Å^−2^, and complete loss of crystallinity was observed at a cumulative dose of 75 e^−^ Å^−2^. Although the stability of the material will vary according to its composition, it has been found that, in contrast to traditional inorganic materials like metals and ceramics, MOFs remain highly sensitive to electron beams and can only withstand very low electron doses—approximately two orders of magnitude lower than those required for traditional HRTEM imaging (Liu *et al.*, 2020 ▸). When considering such a low-dose condition, another decisive factor needs be taken into account—the efficiency of detectors in utilizing electrons that pass through the sample. Early studies on MOF structures using HRTEM imaging used CCD detectors, where the electron signal is converted into a light signal, leading to the degradation of data quality. Thus these studies could only be conducted under conditions far beyond the critical dose, resulting in limited image resolution before structure damage occurred. Only the channels and cages could be observed. However, with technological development, direct detection electron counting (DDEC) cameras have been widely adopted in the field of structural biology owing to their ability to avoid multiple signal conversions. In 2017, DDEC was first applied for low-dose HRTEM imaging of MOF crystals (Zhu *et al.*, 2017 ▸). Under ultra-low electron-dose conditions, combined with accurate image drift correction and contrast transfer function (CTF) correction (Fig. 1 ▸), near-atomic-resolution images of MOFs were obtained with metal clusters and organic ligands distinguishable (Zhang *et al.*, 2018 ▸).

Similarly, the issue of electron utilization efficiency also arises in the structure analysis of MOFs in STEM mode. By collecting electrons at different scattering angles, we can obtain bright-field (BF) images, annular dark-field (ADF) images, high-angle annular dark-field (HAADF) images and integrated differential phase contrast (iDPC) images with various contrast (Fig. 1 ▸). Differently from annular (A)BF images that are sensitive to focus, contrast in HAADF-STEM images is straightforward and related to the square of the atomic number. However, this approach only utilizes a limited proportion of the scattered electrons at high angles, which is very inefficient. To further enhance the efficiency of electron utilization, the iDPC technique with new-generation seg­mented detectors has been developed and has demonstrated significant potential for low-dose imaging of beam-sensitive materials like MOFs. iDPC-STEM enables linear imaging of the projected electrostatic potential, and thereby has a contrast proportional to atomic number that improves the visibility of light elements.

Besides the advancement in detectors, optimizing experimental parameters on the basis of a comprehensive understanding of the electron optics can also significantly improve the electron utilization efficiency for higher signal-to-noise ratio. To strike a balance between signal-to-noise ratio and image resolution, it is crucial to systematically study and identify optimal conditions for imaging MOFs. Convergence angle, collection angle and electron probe current are essential parameters in STEM imaging that have been rarely discussed comprehensively in MOF observation. In our previous study (Wang *et al.*, 2023 ▸), we outlined that setting the inner collection angle equal to the convergence semi-angle gives higher utilization efficiency of electrons, and appropriately decreasing the convergence angle can further enhance the signal-to-noise ratio of the images while maintaining the essential resolution. Under these optimized conditions, crystal-edge features and metal clusters were clearly observed in a MIL-101 crystal, providing a practical reference for low-dose STEM imaging via conventional detectors.

In general, with the development of advanced imaging techniques from various aspects, the electron microscopy characterization of MOF structures has witnessed rapid progress. In this review, we analyse pivotal studies that have advanced the characterization of MOF structures through low-dose imaging methods. Our focus encompasses three critical aspects, including the framework structure, the surface and defects, and the host–guest interactions, crucial for understanding MOFs’ functionalities. At the end of this review, we discuss the current challenges encountered in low-dose (S)TEM imaging of MOFs. Future developments such as four-dimensional scanning transmission electron microscopy (4D-STEM) and the realization of MOF membrane characterization and *in situ* visualization of MOFs are also discussed.

## 2. Low-dose electron microscopy in MOFs

### 2.1. Frameworks

**Figure 2:**
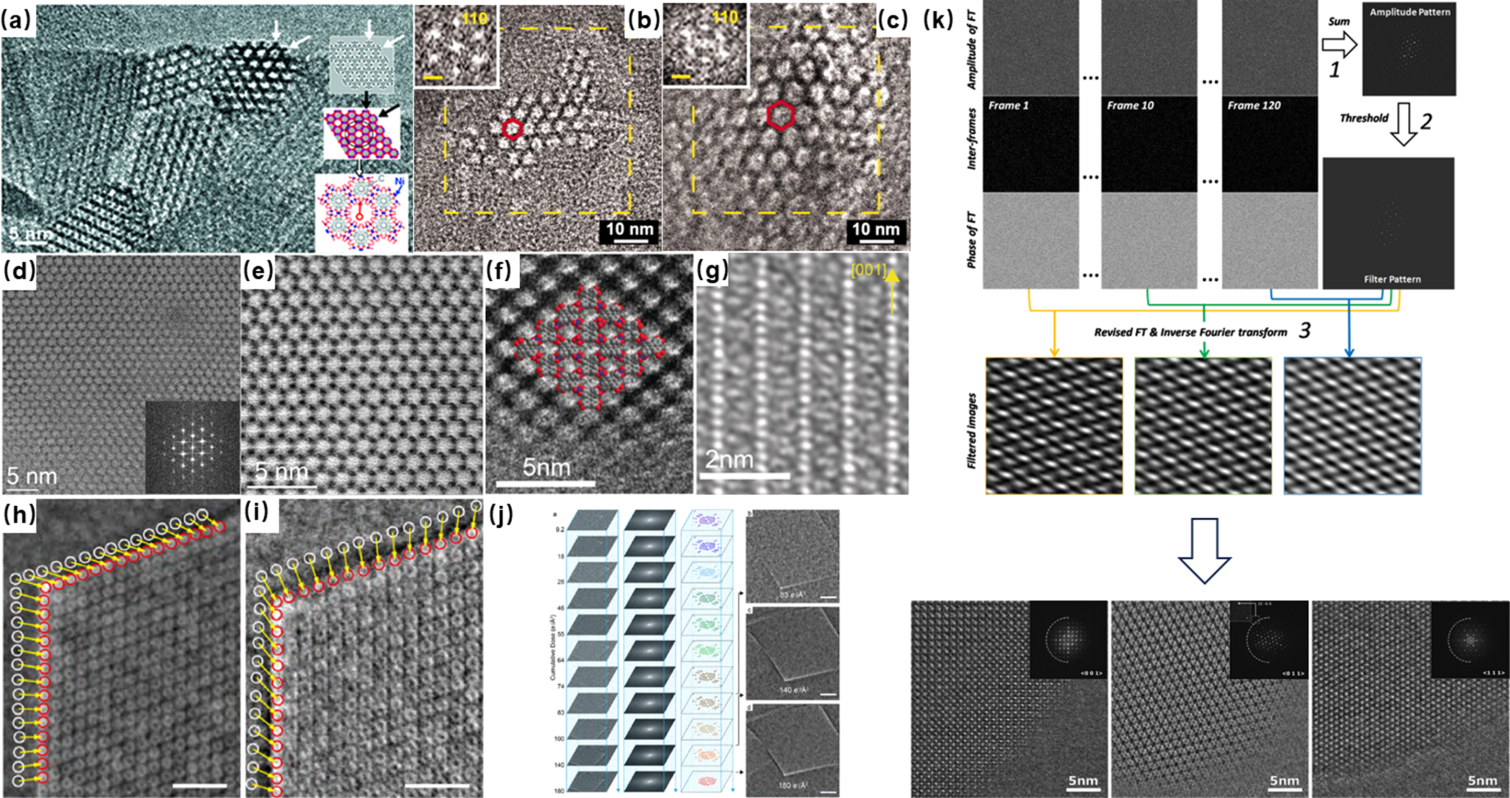
HRTEM images of (*a*) activated Ni-CAT-1 [reprinted with permission from Hmadeh *et al.* (2012 ▸), copyright 2012 American Chemical Society] and (*b*–*c*) IRMOF-74-VII and -IX with the corresponding Fourier diffractogram inset [from Deng *et al.* (2012 ▸), reprinted with permission from AAAS]. (*d*) Cs-corrected STEM-ABF image of Zn-MOF-74 with the Fourier diffractogram inset and (*e*) Fourier filtered image of Zn-MOF-74 with the thermally coloured micrograph [reproduced from the work of Mayoral *et al.* (2015 ▸), copyright 2015 Wiley-VCH Verlag GmbH & Co. KGaA, Weinheim]. (*f*) Zoom-in view of Fourier filtered image of Cu-DBC and (*g*) the image along the [110] zone axis [reproduced from the work of Liu, Zhou *et al.* (2019 ▸), copyright 2020 Wiley-VCH Verlag GmbH & Co. KGaA, Weinheim]. (*h*–*j*) Comparison of the MIL-101 structure under beam irradiation: (*h*) before, (*i*) after and (*j*) the statistical analysis of the Fourier diffractogram with the cumulative electron dose increasing [reprinted with permission from Zhou *et al.* (2020 ▸), copyright 2020 American Chemical Society]. (*k*) Program for alignment of image stacks taken at extremely low dose rates [from Zhang *et al.* (2018 ▸), reprinted with permission from AAAS].

The visualization of pores within MOFs plays a critical role in confirming their topology and intricate structural details. This process is integral to the identification of structures and verifying the accuracy of directional synthesis. In early applications, electron microscopy imaging was constrained to nanoscale resolution because beam damage limited the observation to only the ordered arrangement of pores. Traditional approaches aimed to mitigate electron-beam impact by reducing voltage. Hmadeh *et al.* (2012 ▸) demonstrated this by imaging a two-dimensional MOF, Ni-CAT-1, at 120 kV, confirming its nanoscale-ordered porous structure [Fig. 2 ▸(*a*)]. Similarly, Deng *et al.* (2012 ▸) visualized hexagonally arranged ordered pores in IRMOF-74, employing HRTEM at 120 kV. A ligand-extension strategy enabled variation in pore sizes from 19 to 98 Å in these isostructures, and they observed IRMOF-74-VII and -IX with *d*-spacings of 3.95 and 5.57 nm, respectively [Figs. 2 ▸(*b*) and 2 ▸(*c*)], consistent with X-ray crystallography data. Although Deng *et al.* observed the lattice spacing of MOFs through low-voltage methods, the resolution of the images was not high enough to elucidate finer structural details. The persistent resolution limitations suggested that radiolysis, rather than ‘knock-on’ effects, might be the pre­dominant mechanism of damage. Consequently, researchers shifted towards higher-voltage conditions to counteract radiolysis to enhance the resolution of the image. For instance, Feng *et al.* (2015 ▸) captured HRTEM images of mid-pores in PCN-333 at 200 kV, achieving a resolution of approximately 1 nm, which facilitated the determination of the space group *Fd*3*m*. Although high voltage can mitigate radiolysis, it is more crucial to reduce the electron dose while maintaining a high signal-to-noise ratio of the image. Zhang *et al.* (2018 ▸) developed a simple program to achieve a direct, one-step alignment of the zone axis, incorporating an ‘amplitude filter’ for alignment of image stacks taken at extremely low dose rates [Fig. 2 ▸(*k*)]. This method yielded a series of higher-resolution TEM images of UiO-66 along various axes, allowing for the identification of individual metal atomic columns and benzene rings in the organic linkers after CTF correction of the images. The implementation of low-dose techniques also allows for the recognition of rare interlayer details in a conductive MOF, which is crucial for efficient energy storage. Our research (Liu, Zhou *et al.*, 2019 ▸) revealed that the *c* axis in a new catechol-based MOF measured up to 14 Å as determined from 3D electron diffraction (ED) data. This is unusually large compared with the *c* axis of a typical AA or AB stacking 2D structure. By applying low-dose HRTEM imaging, we confirmed the 1D rhombic channel along the *c* axis and a fourfold interpenetrated 3D structure [Fig. 2 ▸(*f*)]. Additionally, the high-resolution images distinctly showed a 3.6 Å separation between adjacent Cu atom columns and a four-layer periodicity along the [110] direction [Fig. 2 ▸(*g*)]. We concluded that the significant torsion angle between the four benzene rings, resulting from the geometry of the DBC linker, contributed to this interpenetrated structure (Liu, Zhou *et al.*, 2019 ▸).

Compared with TEM images, STEM images are often easier to interpret because they are less influenced by the contrast transfer function, However, the high instantaneous dose rate in STEM presents challenges when imaging MOFs because of potential beam damage. Hmadeh *et al.* (2012 ▸) achieved a milestone by observing MOF structures for the first time using ADF STEM based on low voltage (60 kV) at an aberration-corrected (the DELTA corrector) microscope with a cold-field-emission gun. ADF STEM combined with HRTEM imaging allowed the honeycomb structure of activated Ni-CAT-1 to be clearly observed. Mayoral *et al.* (2015 ▸) used low-dose HADDF STEM at 300 kV to observe Zn-MOF-74. The Zn clusters were clearly identified as bright spots, with the shortest distance identified in the Fourier diffractogram corresponding to the 4–50 spot situated at 2.90 Å. These results proved that STEM can be used not only to observe the arrangement of the pores but also to distinguish the MOF cluster [Figs. 2 ▸(*d*) and 2 ▸(*e*)]. However, the main body of the MOF framework is its organic linker, which is generally composed of carbon, oxygen and nitro­gen, and traditional ADF-STEM methods are not suitable for imaging light elements. Unlike ADF-STEM, the emerging iDPC-STEM technique facilitates the simultaneous imaging of both heavy and light elements by using segmented detectors (Lazić *et al.*, 2016 ▸; Yücelen *et al.*, 2018 ▸). Several studies have reported the high-resolution stationary imaging of MIL-101 by this technique at low-dose conditions. This can directly reveal the structural details of the organic framework, including the super-tetrahedron. In addition to high signal-to-noise ratio imaging at controlled low dose, the iDPC technique can also achieve a higher resolution because of its extraordinary electron utilization efficiency. iDPC images have also resolved the coordination of Cr nodes and organic linkers inside the frameworks with an information transfer of ~1.8 Å, which demonstrated the outstanding capacity of the iDPC technique for the imaging of beam-sensitive materials and light-element species (Shen *et al.*, 2020 ▸). On the basis of these advantages, our study focused on the dynamic response of the MIL-101 local structure when exposed to an electron beam during STEM imaging. Zhou *et al.* (2020 ▸) investigated the relationship between cell parameters and cumulative electron dose, observing a continuously anisotropic decrease across different crystallographic directions even at low-dose conditions [Figs. 2 ▸(*h*) and 2 ▸(*i*)]. Specifically, when the electron dose increases, the (111) lattice plane shrinks faster, as revealed by statistical analysis, and the local evolvement was found to be dependent on both the lattice plane and the specific position in the crystal. This investigation highlights the potential of iDPC-STEM for detailed spatial and temporal analysis of the structural evolution of MOFs under various dose conditions.

### 2.2. Surfaces and defects

**Figure 3:**
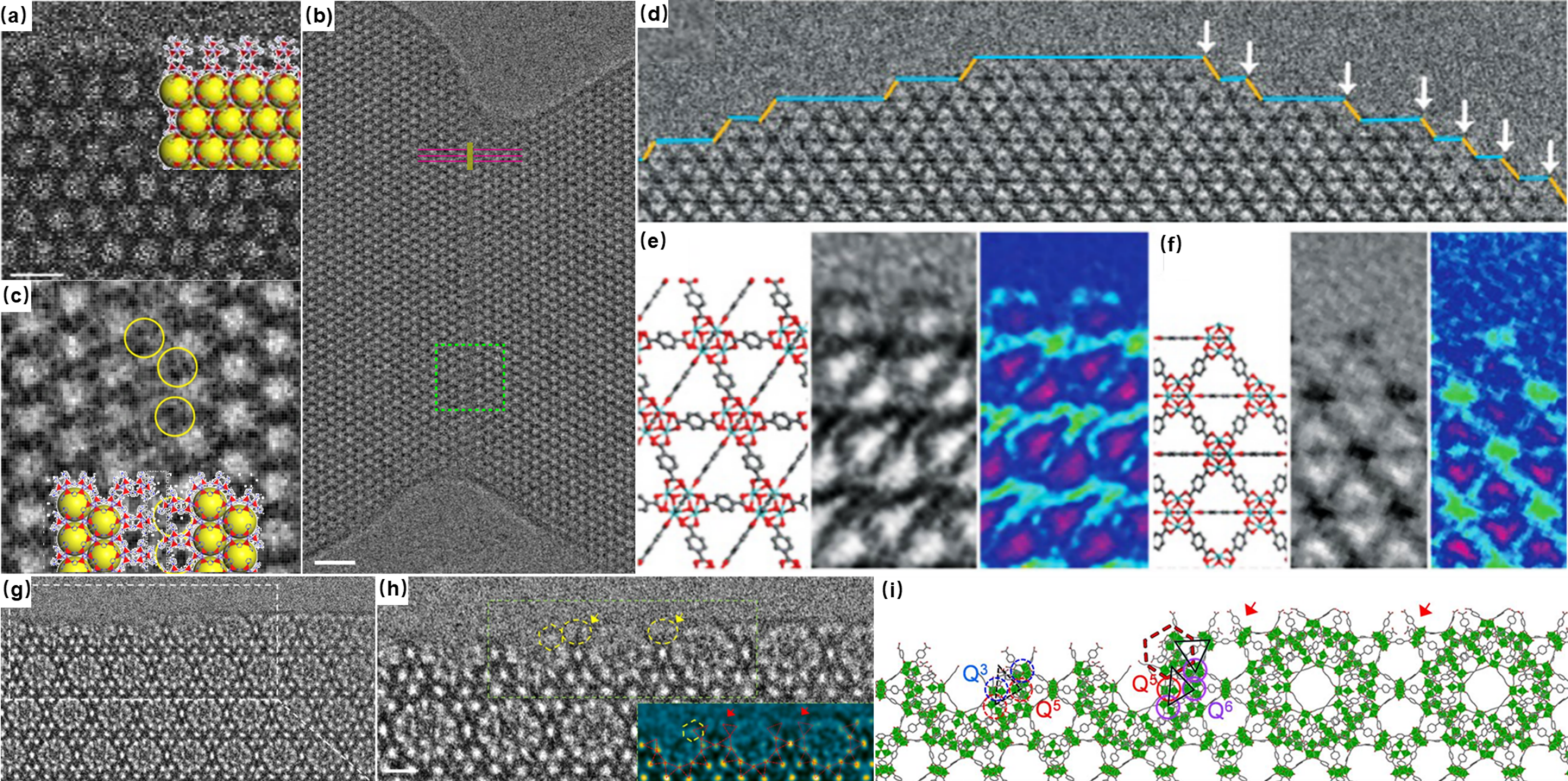
(*a*) HRTEM image of ZIF-8 taken along the [111] axis incorporated with the structure model, (*b*) the interface of ZIF-8 and (*c*) the CTF-corrected HRTEM image of the region marked with a green square in (*b*) [reproduced from the work of Zhu *et al.* (2017 ▸), with permission from Springer Nature]. (*d*–*f*) Ligand-terminated {111} surface and metal-terminated {100}/{111} kink: (left) structural model; (middle) processed HRTEM image; (right) the image in rainbow colours to increase the visibility of the ligand contrast [from the work of Zhang *et al.* (2018 ▸), reprinted with permission from AAAS]. (*g*–*i*) The transition from a sublayer surface to a stable surface: (*h*) zoom-in image from (*g*) (a coloured image was used to increase the contrast of chromium trimers) and (i) the structure model of the transition [reproduced from the work of Han *et al.* (2020 ▸), copyright 2020 Wiley-VCH GmbH].

MOF nanocrystals inherently display a variety of local and aperiodic structural features, including surfaces, interfaces and defects. These features also have a great impact on the MOF’s practical application. However, capturing the intricate details of the local structure before it is damaged by electrons remains a significant challenge. Hmadeh *et al.* (2012 ▸) reported the first observation of MOF surfaces. However, limited resolution hindered the acquisition of more detailed data for matching the simulated images. Nevertheless, with the advancements in low-dose techniques, local structures could be observed with higher signal-to-noise ratios. Zhu *et al.* (2017 ▸), for the first time, implemented a DDEC camera to perform low-dose high-resolution imaging of ZIF-8 crystals. They proposed that the (110) surface may exhibit ‘zigzag’ or ‘armchair’-type terminations. By observing the exposed (110) surface from the [111] incidence direction and comparing with the simulated images in Fig. 3 ▸(*a*), they confirmed the armchair-type surface terminations. However, due to the large defocus value (~550 nm) used in non-Cs-corrected TEM (where Cs relates to spherical aberration), their images suffered from contrast delocalization and pronounced Fresnel fringes, inhibiting a more detailed structural analysis of the topmost Zn layer. Subsequently, in an effort to obtain higher-resolution images of the surface, Zhang *et al.* (2018 ▸) utilized a series of low-dose techniques to observe different coexisting surface termination modes in UiO-66 crystals [Fig. 3 ▸(*d*)]. They noted that the majority of the exposed {111} crystal surface was terminated by benzene-1,4-di­carboxyl­ate (BDC) linkers. In contrast, the small truncation surface {111} and {100} faces were terminated by Zr clusters [Figs. 3 ▸(*e*) and 3 ▸(*f*)] . This work revealed distinct termination of organic linkers and metal clusters on the surface through the enhanced resolution. Similar phenomena with organic ligands as terminators can also be observed in conductive MOFs by carefully adjusting the focus under low-dose conditions (Liu *et al.*, 2021 ▸; Liu, Chen *et al.*, 2019 ▸). Alternatively, the delocalization effect on surfaces caused by inherent spherical aberration can be diminished effectively by minimizing the Cs value. Meanwhile, combining small Cs with appropriate defocus can directly present the atomic/molecular column as bright contrast in images according to the charge density projection approximation, facilitating a more straightforward interpretation of HRTEM images. On the basis of this theory, Han *et al.* (2020 ▸) obtained HRTEM images of MIL-101 structures which are drift corrected and taken with a slight overfocus in conjunction with a negative and small Cs for enhancing image contrast. The result demonstrated the structures of sublayer surfaces and their evolution to stable surfaces regulated by inorganic Cr_3_(μ_3_-O) trimers, providing a method to observe growth of the MOFs via the assembly of sublayer surfaces [Figs. 3 ▸(*g*)–3 ▸(*i*)].

In addition to the TEM imaging technique, the development of the low-dose STEM imaging method is also critical. The aforementioned iDPC-STEM imaging utilizes a new segmented detector, which, although effective, tends to be costly and infrequently available. Therefore, it is important to discuss and develop a general approach for traditional STEM imaging at low-dose conditions. In a recent study, Wang *et al.* (2023 ▸) systematically studied the relationship between convergence/collection angle and signal-to-noise ratio and image resolution. They proposed to calculate and design the optimal experimental parameters (convergence angle and collection angel) according to requirements. The experiment involved continuous adjustment of condenser and intermediate lenses in a commercial transmission electron microscope. A smooth edge along the (111) facet, four half mesopore I pores along the (100) facet and surface steps can be visualized unambiguously in a MIL-101 crystal. This study provides a powerful method for conventional STEM imaging of MOF materials with improved signal-to-noise ratio and resolution.

Building upon the understanding with surfaces of MOFs, we can also investigate the formation of interfaces between crystals, which is crucial for the diverse assemblies and agglomerations of MOFs. Zhu *et al.* (2017 ▸) used HRTEM imaging for the first time to investigate the interface of MOFs. They observed the directional self-assembly of ZIF-8 crystals via (110) surfaces. Fig. 3 ▸(*a*) shows two ZIF-8 crystals connected through a coherent interface, exhibiting an arm-to-notch configuration. However, unlike perfect crystal surfaces, the crystal structure is disrupted by the existence of an additional layer of ligands along the [111] direction. Furthermore, molecular dynamics simulations have shown that the introduction of interfacial cavities significantly enhances the diffusion coefficients of guest molecules across various loading conditions. These observations are helpful to understand how ZIF-8 crystals self-assemble and the subsequent influence of interfacial cavities on mass transport of guest molecules.

**Figure 4:**
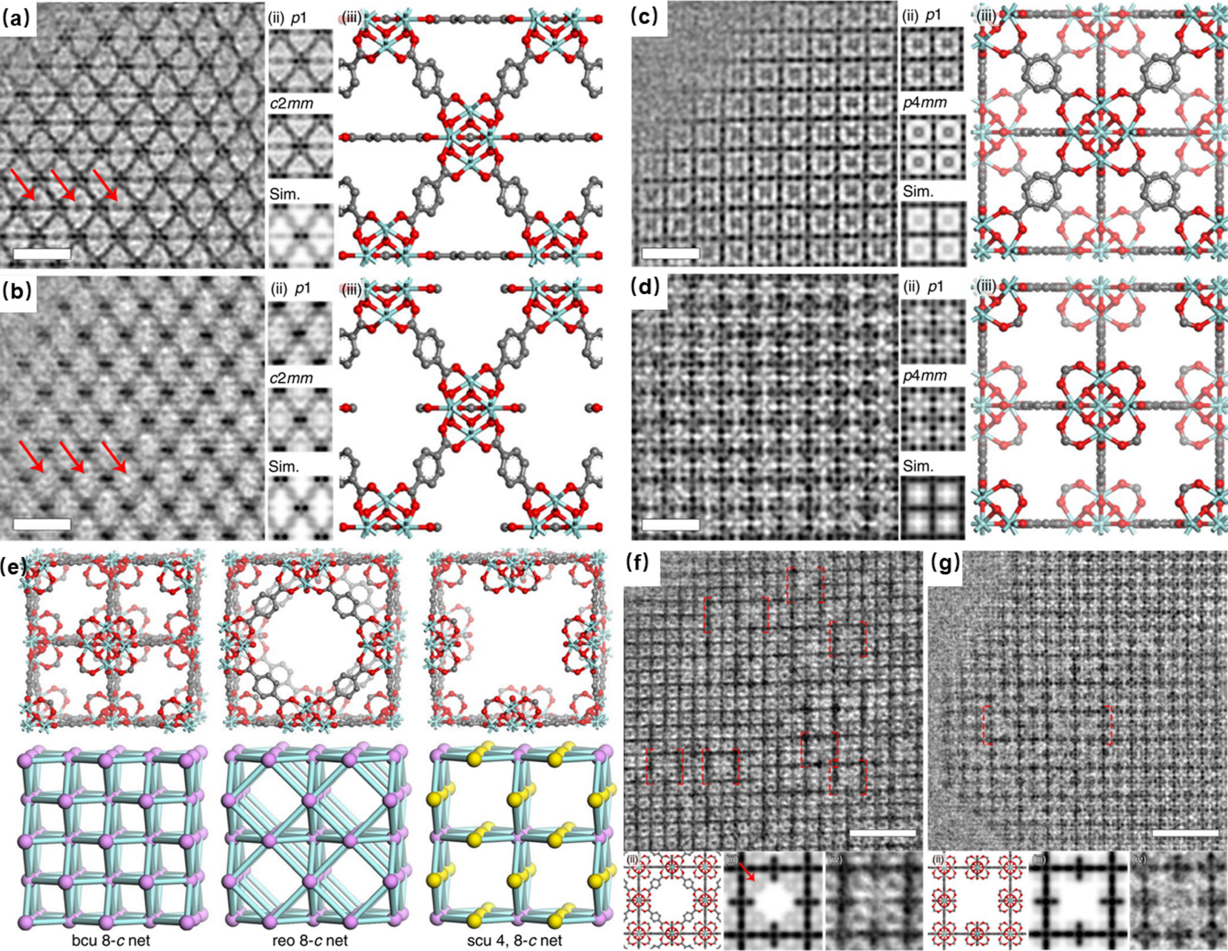
(*a*–*d*) CTF-corrected HRTEM images and structural models along the [110] zone axis of (*a*) perfect UiO-66 and (*b*) a defective region, and along the [110] zone axis of (*c*) perfect UiO-66 and (*d*) a defective region. (*e*) Three different defects and their structure models. (*f*–*g*) CTF-corrected images along the [001] direction showing the defect distribution of the **reo** structure (*f*) and the **scu** structure (*g*). [Reproduced with permission from Springer Nature from the work of Liu, Chen *et al.* (2019 ▸).]

Beyond surface and interface analysis, defects also exist widely in different kinds of crystalline materials, as well as MOFs. Characterizing these defects is vital for performance optimization of MOF materials, as it is helpful to realize directional defect engineering which can lead to the creation of both open metal sites and targeted adjustments in porosity. The unique framework structure of MOFs results in various types of defects, including the absence of linkers and clusters. UiO-66 has been extensively studied and reported for missing linkers in its structure. Traditional diffraction methods are limited to characterizing MOFs in an averaged manner, lacking the precision to determine specific defect distributions or locations within the MOF structure. In contrast, low-dose HRTEM imaging has enabled the direct observation of structural defects in MOFs at atomic resolution, significantly advancing our understanding of these materials. Liu, Chen *et al.* (2019 ▸) reported a systematic investigation of internal defects of UiO-66. They synthesized a series of defective UiO-66 structures using formic acid as a modulator and observed the absence of linkers along the [001] and [110] directions. Using Fourier summation of the crystal structure factors determined from the HRTEM images in Figs. 4 ▸(*a*)–4 ▸(*d*), they successfully constructed a three-dimensional potential map. This map illustrated the observed defect structure, clearly depicting the Zr_6_O_8_ clusters and BDC linkers, and also revealing the terminal formate ligands that replace the absent BDC ligands to cap the open metal sites. This study also proposed the intriguing possibility of manipulating defect surface groups by altering experimental conditions, suggesting a new avenue for tailoring properties of MOFs. On the basis of this study, Liu *et al.* also discovered that, as well as the **bcu** topology characterized by missing-linker structures, there are two other types of topologies, **reo** and **scu**, exhibiting missing-cluster structures. The **reo** structure is obtained by removing an octagon-shaped Zr_6_O_8_ cluster from the perfect **fcu** structure, while **scu**, being the structure with the most significant defect, corresponds to the removal of an octagon-shaped Zr_6_O_8_ cluster from the **bcu** structure [Fig. 4 ▸(*e*)]. In addition, it has been observed that missing-linker defects are more prevalent, while missing-cluster defects are confined to smaller regions, spanning only a few unit-cell dimensions [Figs. 4 ▸(*f*) and 4 ▸(*g*)]. For the first time, HRTEM techniques have enabled the identification of multiple coexisting defects within a single MOF crystal, marking a departure from previous research which primarily focused on individual defects. This investigation provides insights into the potential relationships between these defects and the evolution of crystal structures, opening new pathways for directional defect engineering in MOFs.

### 2.3. Guest and host

**Figure 5:**
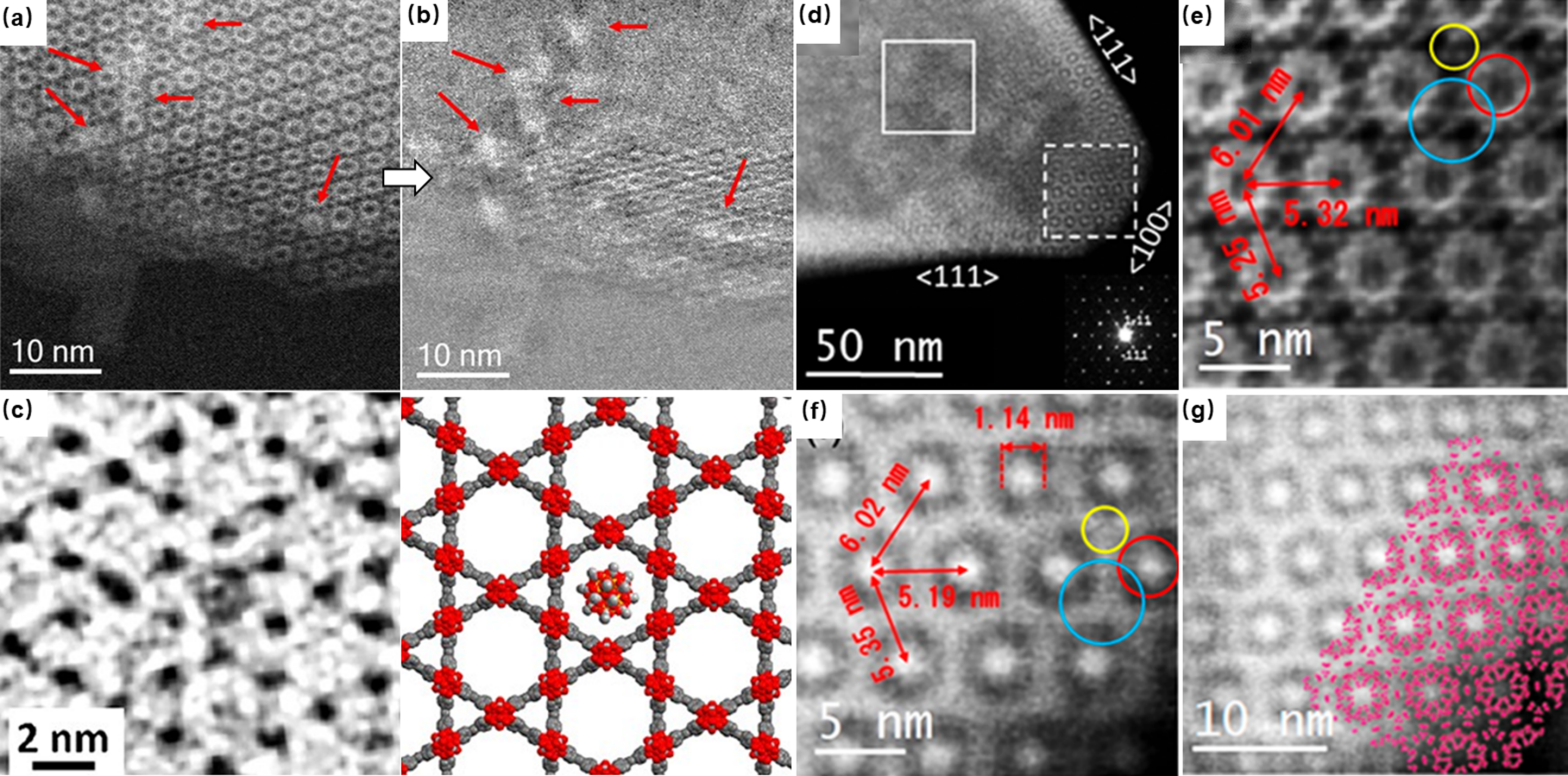
(*a*) High-magnification image of MIL-100(Fe) with stronger contrast indicated by red arrows and (*b*) visualization of the Ag particles with the periodic arrangement removed [reproduced from the work of Mayoral *et al.* (2017 ▸), copyright 2017 Wiley-VCH Verlag GmbH & Co. KGaA, Weinheim]. (*c*) Image of Mn_12_Ac@NU-1000 and its structure model [reprinted with permission from Aulakh *et al.* (2019 ▸), copyright 2019 American Chemical Society]. (*d*–*g*) HAADF-STEM images of CsPbI_3_@MIL-101. The magnified images (*e*) and (*f*) correspond to the solid and dashed boxes in (*d*); the MIL-101 framework along the [110] direction is overlaid in (*g*) [reprinted with permission from Cha *et al.* (2019 ▸), copyright 2019 American Chemical Society].

A deeper understanding of the local structures of MOFs opens up opportunities for their application across various fields. The large surface area and high porosity characteristic of MOFs allow for the adjustment of pore size and shape, enabling selective adsorption of guests to tailor the microenvironments for specific functionality. Investigating the relationship between the host (MOF) and the guest (incorporated molecules/clusters/nanoparticles) is crucial for comprehending the underlying mechanisms that affect the properties of composite materials. However, traditional diffraction analysis methods face challenges in accurately locating guest molecules, mainly due to the lack of periodic distribution of guest molecules in the MOF. Although indirect methods like thermogravimetric analysis can confirm the presence of guest molecules within the MOF framework, the precise locations of these guest molecules with regard to different pores/channels remain elusive (Liu *et al.*, 2020 ▸). Electron microscopy imaging shows great advantages in local structure analysis, but a significant problem arises when imaging MOF systems. The primary issue is the need to simultaneously image both the guests within the MOF and the MOF framework itself, especially considering the high sensitivity of MOFs to electron beams. Recent advances in low-dose TEM imaging techniques represent a significant breakthrough in overcoming these difficulties, providing more direct and precise insights into the spatial distribution of the guest. In an initial study, Mayoral *et al.* (2017 ▸) successfully utilized HAADF-STEM to simultaneously image silver nanoparticles and the framework structure of MOFs. However, the image contrast suggested that most of the silver resided on the surface of the MOFs, rather than being uniformly distributed [Figs. 5 ▸(*a*) and 5 ▸(*b*)]. Subsequently, another study achieved a significant breakthrough by using low-dose TEM to precisely visualize the encapsulation and coordination of Mn_12_Ac clusters within the hexagonal channels of the MOF NU-1000 [Fig. 5 ▸(*c*)]. Despite this advancement, the interpretation of the TEM images remains contentious due to the phenomenon of contrast inversion, casting some uncertainty on the observed results (Aulakh *et al.*, 2019 ▸). Subsequently, Cha *et al.* (2019 ▸) successfully synthesized the all-inorganic lead halide perov­skites directly within the pores of MIL-101 through a simple two-step solution-based *in situ* method. HAADF-STEM images with more straightforward contrast revealed the presence of CsPbI_3_ quantum dots within the mesoporous cages of MIL-101 as the quantum dots exhibited remarkably bright contrast [Figs. 5 ▸(*d*)–5 ▸(*g*)]. This study demonstrated the potential of MIL-101 as a micro-reaction vessel for synthesizing stable and homogeneous perovskite quantum dots.

**Figure 6:**
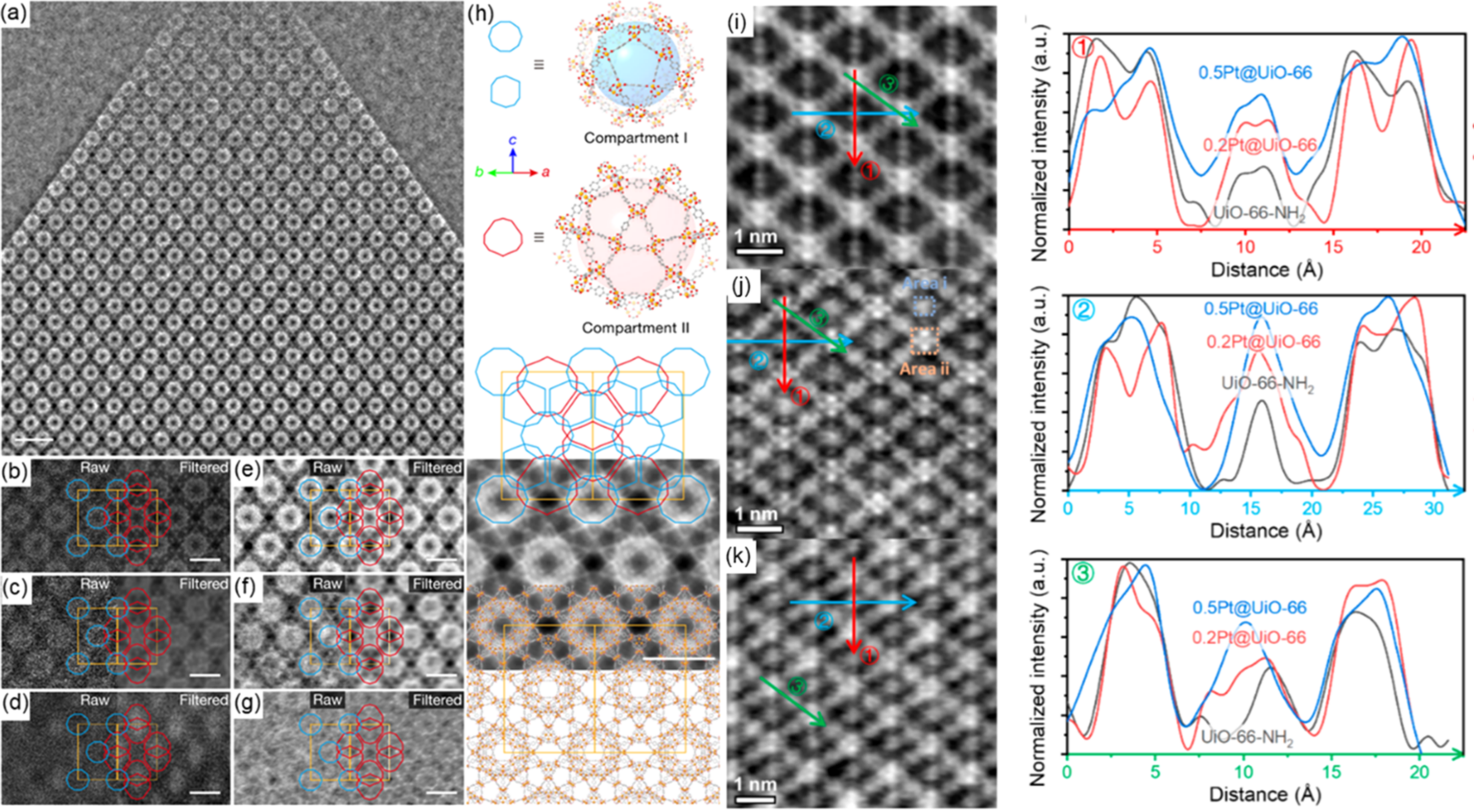
(*a*) iDPC-STEM images of TiO_2_-in-MOF sample taken from [110] incidence, (*b*, *e*) pure MIL-101-Cr, (*c*, *f*) 23%-TiO_2_-in-MIL-101-Cr and (*d*, *g*) 42%-TiO_2_-in-MIL-101-Cr, and (*h*) illustration of two types of mesopore [reproduced with permission from Springer Nature from the work of Jiang *et al.* (2020 ▸)]. (*i*–*k*) iDPC-STEM images of Pt encapsulated in the UiO-66 framework: (*i*) pure UiO-66-NH_2_, (*j*) 0.2Pt@UiO-66 and (*k*) 0.5Pt@UiO-66 with normalized intensity profiles along different axes [reprinted with permission from Liu, Chen *et al.* (2023 ▸), copyright 2023 American Chemical Society].

Previous researchers have successfully uncovered the presence of guests within MOF pores. However, owing to limitations in resolution, signal-to-noise ratio and the methodologies employed, these studies fell short in elucidating how these guests are distributed across different types of pores within the MOFs. Recent advancements in electron microscopy technology have partially addressed these issues. Jiang *et al.* (2020 ▸), in their investigation of the photocatalytic properties of TiO_2_-in-MOF composites, achieved precise localization of TiO_2_ nanoparticles within different MOF mesopores when incorporating TiO_2_ into MIL-101-Cr MOF crystals. Enabled by the high contrast and high resolution of iDPC and simultaneous HADDF-STEM images, quantitative analysis of image contrast revealed the presence of TiO_2_ nanoparticles in each individual pore of the MOF, as well as their filling behaviour in different types of pores at varying TiO_2_ filling amounts [Figs. 6 ▸(*a*) and 6 ▸(*h*)]. For instance, it was observed that the nanoparticles were present only in mesopore I in a 23%-TiO_2_-in-MIL-101-Cr sample [Figs. 6 ▸(*c*) and 6 ▸(*f*)], whereas in a 42%-TiO_2_-in-MIL-101-Cr sample [Figs. 6 ▸(*d*) and 6 ▸(*g*)], TiO_2_ was found in both mesopore I and mesopore II. This analysis also facilitated a comparison of photocatalytic efficiency among composites with varying locations and amount of TiO_2_. With advancements in low-dose imaging techniques, atomic bonding in MOF composites can be explored with greater precision. Liu, Chen *et al.* (2023 ▸) utilized low-dose iDPC-STEM imaging to observe the atomic details of Pt_1_@UiO-66 and Pd_1_@UiO-66-NH_2_ systems. In the Pt@UiO-66 system, a platinum atom was located on the benzene ring of the p-BDC linker. Conversely, in the Pd@UiO-66-NH_2_ system, a single palladium atom attached to the amino group was observed [Figs. 6 ▸(*i*)–6 ▸(*k*)]. This study not only provides valuable insights into the specific adsorption sites of individual metal atoms within the UiO-66 framework but also sheds light on the intricate interactions between single metal atoms and the structure of MOFs. In the same study, obvious agglomerated metal clusters were observed in the Pt@UiO-66-NH_2_ and Pd@UiO-66 systems. Combined with theoretical calculations, this observation demonstrates that amino groups do not always facilitate the formation of single-atom catalysts. Collectively, these studies underscore the pivotal role of low-dose EM imaging in providing conclusive evidence of guest molecules/clusters/nanoparticles within the framework of MOFs, especially in revealing intricate details about the spatial arrangement of guests while maintaining the integrity of the MOF structure.

## 3. Summary and outlook

With the advancement of instrumentation and the development of methodology, low-dose high-resolution imaging technology has been rapidly developed. In the preceding text, we have summarized several notable studies that demonstrate this progress. That work encompasses a range of topics, including the characterization of framework structures of MOFs, the analysis of defects and surface/interface structures, and the investigation of the distribution of guest molecules/clusters/ nanoparticles within the MOF framework. Overall, low-dose high-resolution imaging has allowed significant advances in the characterization of MOF materials. However, some challenges remain, such as the resolution still being insufficient to distinguish each individual atom, and further improvement is needed. Additionally, current TEM imaging predominantly focuses on two-dimensional projection image analysis, lacking the detailed three-dimensional information that is extremely important for composite materials, particularly in terms of guest distribution within MOF frameworks. Furthermore, traditional sample preparation methods for inorganic materials are not entirely applicable to soft materials like MOFs, while sample preparation is also crucial for TEM analysis. Therefore, we consider some prospects for these challenges in the following.

Recent improvements in pixelated detectors, characterized by high speed and high detection quantum efficiency, have significantly advanced the development of 4D-STEM. A key application of this technique is electron ptychography, which reconstructs the transmission function from the convergent beam electron diffraction data in 4D-STEM to accurately determine sample structures. Utilizing complex algorithms, electron ptychography effectively negates spherical aberration effects, achieving sub-Å resolution. Notably, a recent study demonstrated that imaging resolution with uncorrected STEM could reach an exceptional 0.44 Å, surpassing the resolutions typically achieved with aberration-corrected instruments (Nguyen *et al.*, 2024 ▸). 4D-STEM, by collecting a more comprehensive fraction of signals, offers enhanced electron utilization efficiency compared with traditional STEM imaging techniques, making it particularly suitable for imaging materials sensitive to electron beams. Moreover, this technique facilitates the acquisition of atomic-resolution images without the necessity for precisely focused electron beams, which lowers the requirements for imaging conditions.

**Figure 7:**
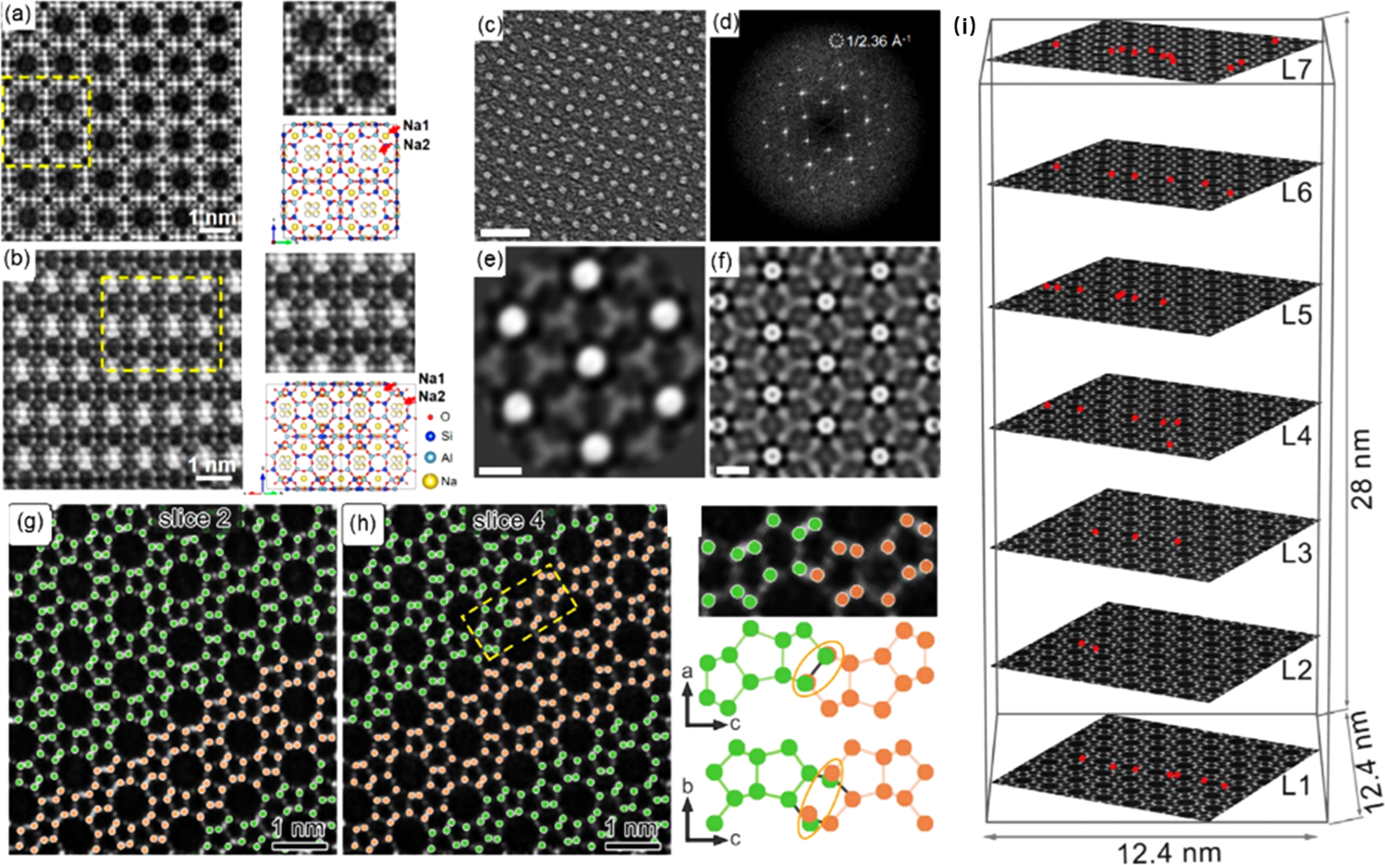
(*a*–*b*) Reconstructed images of Na-LTA based on the ePIE algorithm along the [100] and [110] zone axes with structural models [reprinted with permission from Dong *et al.* (2023 ▸), copyright 2023 American Chemical Society]. (*c*–*d*) Ptychographic reconstruction of MOLs from 4D-STEM data with information transfer to 1/2.36 Å^−1^, (*e*) an image showing Hf clusters and BTB ligands, and (*f*) ptychographic reconstruction of a simulated dataset of Hf MOLs with four layers [reproduced from the work of Peng *et al.* (2022 ▸) under a Creative Commons Attribution 4.0 International License]. (*g*–*h*) Distinction between coexisting MFI and MEL phases, and (*i*) identification of O vacancies in ZSM-5 [from Zhang *et al.* (2023 ▸), reprinted with permission from AAAS].

In order to further explore the application of electron ptychography in electron-beam-sensitive materials, Dong *et al.* (2023 ▸) implemented the extended ptychographic iterative engine (ePIE) algorithm to investigate a typical electron-beam-sensitive zeolite (Na-LTA, Si/Al ≃ 1). The four-dimensional data were reconstructed to obtain high-resolution, high signal-to-noise ratio images along the [100] and [110] axes [Figs. 7 ▸(*a*) and 7 ▸(*b*)]. The results clearly revealed all the framework atoms of Na-LTA in atomic resolution, including oxygen. Meanwhile, the extra-framework Na^+^ was also observed, as well as its inhomogeneous contrasts within the S8Rs which might be related to 1/4 occupancy. While notable advancements have been achieved in zeolite imaging, the application of ptychography techniques in the more beam-sensitive MOF systems is rare. Metal–organic layers (MOLs), with their ultra-thin characteristics, present an ideal candidate for ptychography observation [Figs. 7 ▸(*c*) and 7 ▸(*d*)]. Peng *et al.* (2022 ▸) utilized electron ptychography at 80 keV to successfully discern individual Hf clusters and their connecting ligands [Fig. 7 ▸(*f*)]. The resulting images revealed a two-dimensional network structure, where each Hf cluster is encircled by six ligands and each ligand is linked to three clusters [Fig. 7 ▸(*e*)]. This significant finding opens avenues for further exploration into 2D and potentially 3D MOF framework structures at higher resolution on an atomic scale.

In addition, through the multi-slice method, ptychography can provide depth information. This solves the limitation of traditional STEM technology, which usually displays two-dimensional projection information. Zhang *et al.* (2023 ▸) used low-dose 4D-STEM to collect data on ZSM-5 zeolite, and adopted an iterative algorithm combined with the multi-slice method. The resolution of the reconstructed data reached 0.85 Å. The obtained images not only precisely locate all atom positions of the zeolite [Fig. 7 ▸(*i*)] but, at the same time, provide a depth resolution of about 6.6 nm. This enabled the visualization of the zeolite intergrowth structure in three-dimensional space and facilitated the distinction between coexisting MFI and MEL phases [Figs. 7 ▸(*g*) and 7 ▸(*h*)]. This study provides a feasible approach for collecting three-dimensional structural information of electron-beam-sensitive materials, marking a significant step forward in the field.

For structural characterization of a MOF crystal, in order to obtain more accurate information from a (S)TEM image, we not only must make efforts in hardware and software but also need to further develop the sample preparation. Owing to the influence of the dynamical effect, obtaining detailed structural information is more feasible with thinner samples. The imaging of most MOF crystals is predominantly confined to the nanoscale because of sample thickness constraints. Therefore, imaging larger MOF crystals presents significant challenges in the field. Traditional methods like crushing, grinding and slicing would easily damage their structure. Moreover, the random orientation of small crystals makes it challenging to locate the specific crystal lattices required for analysis. Focused ion beam (FIB) is a highly effective technique for preparing TEM samples, particularly favoured in the study of metal materials. Its distinct advantage lies in its capability to prepare thin samples from specific areas of large crystals with precise orientation control. Building on this technique, Zhou *et al.* (2022 ▸) implemented a cryogenic FIB method for preparing large-area high-quality specimens from bulk MOF crystals with the desired orientations. This advancement significantly reduces the time required for crystal searching and orientation alignment, thereby conserving the electron dose necessary for high-resolution imaging. It represents a novel approach for preparing samples from large crystals for low-dose HRTEM imaging, particularly beneficial for electron-beam-sensitive materials. Additionally, the cryogenic method not only preserves the crystallinity during sample preparation but also effectively immobilizes solvent or gas molecules within the pores of the MOF. Utilizing a plunge-frozen method, Sun *et al.* (2019 ▸) studied the crystal structure of COF-300 upon H_2_O adsorption and elucidated unambiguously the location of guest molecules in the pores. Similarly, Ling *et al.* (2022 ▸) observed structural changes in the MOF MIL-53 upon water adsorption into pores using cryo-3D ED, and extended the methodology to observe the dynamic changes of the MIL-53 skeleton under *in situ* liquid phase and *in situ* gas phase conditions. However, all these studies reveal the average structure of MOF crystals through diffraction, and how to reveal the uneven distribution of gas or liquid molecules in the framework under *in situ* conditions through imaging should be an important future research direction.

The current advancements in MOF membrane preparation methods have found widespread applications in gas separation and adsorption fields. But the structural characterization of the MOF membrane is rarely attempted with TEM analysis due to the challenges in preparing TEM samples, which are often difficult to control in terms of thickness and size. In recent research, Liu, Miao *et al.* (2023 ▸) employed an ultra-thin precursor mixture method to interface with the underlying crystalline substrate, resulting in a ZIF membrane that possesses a thickness as low as a single structural building unit (2 nm). This technique enables the production of MOF membranes with a single cell thickness, which are unattainable with traditional thinning methods. The potential of using HR-TEM in characterizing the structural intricacies of the MOF membrane is demonstrated. All the field mentioned above show that HR-(S)TEM till holds significant potential for broad applications in characterization of MOF systems.
